# Dihydromyricetin ameliorates liver fibrosis via inhibition of hepatic stellate cells by inducing autophagy and natural killer cell-mediated killing effect

**DOI:** 10.1186/s12986-021-00589-6

**Published:** 2021-06-19

**Authors:** Xi Zhou, Li Yu, Min Zhou, Pengfei Hou, Long Yi, Mantian Mi

**Affiliations:** Research Center for Nutrition and Food Safety, Chongqing Key Laboratory of Nutrition and Food Safety, Institute of Military Preventive Medicine, Third Military Medical University (Army Medical University), NO. 30th Gao Tan Yan Street, Shapingba District, Chongqing, 400038 People’s Republic of China

**Keywords:** Dihydromyricetin, Natural killer cells, Hepatic stellate cells, Liver fibrosis, IFN-γ

## Abstract

**Background:**

This study investigated the mechanisms underlying the preventive effect of dihydromyricetin (DHM) against liver fibrosis involving hepatic stellate cells (HSCs) and hepatic natural killer (NK) cells.

**Methods:**

A carbon tetrachloride (CCl_4_)-induced liver fibrosis model was established in C57BL/6 mice to study the antifibrotic effect of DHM based on serum biochemical parameters, histological and immunofluorescence stainings, and the expression of several fibrosis-related markers. Based on the immunoregulatory role of DHM, the effect of DHM on NK cell activation ex vivo was evaluated by flow cytometry. Then, we investigated whether DHM-induced autophagy was involved in HSCs inactivation using enzyme-linked immunosorbent assays, transmission electron microscopy, and western blot analysis. Thereafter, the role of DHM in NK cell-mediated killing was studied by in vitro coculture of NK cells and HSCs, with subsequent analysis by flow cytometry. Finally, the mechanism by which DHM regulates NK cells was studied by western blot analysis.

**Results:**

DHM ameliorated liver fibrosis in C57BL/6 mice, as characterized by decreased serum alanine transaminase and aspartate transaminase levels, decreased expressions of collagen I alpha 1 (CoL-1α1), collagen I alpha 2 (CoL-1α2), tissue inhibitor of metalloproteinases 1 (TIMP-1), α-smooth muscle actin (α-SMA) and desmin, as well as increased expression of matrix metalloproteinase 1 (MMP1). Interestingly, HSCs activation was significantly inhibited by DHM in vivo and in vitro. As expected, DHM also upregulated autophagy-related indicators in liver from CCl_4_-treated mice. DHM also prevented TGF-β1-induced activation of HSCs in vitro by initiating autophagic flux. In contrast, the autophagy inhibitor 3-methyladenine markedly abolished the antifibrotic effect of DHM. Surprisingly, the frequency of activated intrahepatic NK cells was significantly elevated by DHM ex vivo. Furthermore, DHM enhanced NK cell-mediated killing of HSCs by increasing IFN-γ expression, which was abolished by an anti-IFN-γ neutralizing antibody. Mechanistically, DHM-induced IFN-γ expression was through AhR-NF-κB/STAT3 pathway in NK cells.

**Conclusion:**

These results demonstrated that DHM can ameliorate the progression of liver fibrosis and inhibition of HSCs activation by inducing autophagy and enhancing NK cell-mediated killing through the AhR-NF-κB/STAT3-IFN-γ signaling pathway, providing new insights into the preventive role of DHM in liver fibrosis.

**Supplementary Information:**

The online version contains supplementary material available at 10.1186/s12986-021-00589-6.

## Background

Liver fibrosis, which is a dynamic progress of cellular and molecular activities characterized by excessive deposition of extracellular matrix (ECM) molecules, is believed to be a critical step in the progression of liver disease. Persisting fibrosis is generally documented as the initiating factor of liver cirrhosis and, ultimately, even hepatic cancer. The worldwide epidemic and continuously increasing prevalence of hepatic fibrosis constitute an enormous public health challenge [1, 2]. However, there is still no approved drug to treat liver fibrosis, and most liver disease patients have low compliance with physical exercise [3]. Hence, novel preventive and therapeutic strategies are urgently needed to manage liver fibrosis.

Over the past decades, several pharmacological treatments, such as vitamin E, metformin, lipid-lowering agents, and insulin sensitizers, have been explored for liver disease [4, 5]. Unfortunately, none of these treatments showed significant efficacy or long-term safety in animal experiments and cellular studies. Recently, an increasing number of studies have suggested that natural polyphenols are able to inhibit various kinds of metabolic disorders [6]. These polyphenols are easily obtainable from food and can be absorbed with relatively few side effects. Dihydromyricetin (DHM), the most abundant flavonoid in *Ampelopsis grossedentata*, comprises over 30% of the dry weight of the leaves and stems of vine tea [7]. Previous studies demonstrated that DHM has potential anti-inflammatory, antioxidative properties, as well as hepatoprotective, lipid regulatory, and antitumor effects [8, 9]. Our previous studies also showed that DHM supplementation improved lipid and glucose metabolism and ameliorated nonalcoholic fatty liver disease (NAFLD) by enhancing mitochondrial respiration while maintaining redox homeostasis [10]. A randomized controlled trial further indicated that DHM supplementation significantly decreased hepatic steatosis and suppressed inflammation caused by NAFLD [11]. DHM has also been reported to increase resistance to the fibrosis-induced agent carbon tetrachloride (CCl_4_) [12], indicating the prospective use of DHM for alleviation of liver fibrosis. However, there is no report about its underlying mechanism.

Activated hepatic stellate cells (HSCs) play a vital role in the development of liver fibrosis. In the normal liver, quiescent HSCs maintain a nonproliferative phenotype and are responsible for the storage of vitamin A. When the liver is exposed to hazards, quiescent HSCs switch to the activated phenotype, characterized by a decline in vitamin A droplets and the proliferation of ECM-enriched myofibroblasts, which ultimately causes liver fibrosis [13]. Thus, suppressing or eliminating HSCs activation could be a potential therapeutic antifibrotic therapeutic strategy for liver fibrosis. On the one hand, studies have provided specific evidence to pinpoint the fundamental role of autophagy in HSCs activation [14]. Some natural polyphenols exhibit high efficiency in triggering the autophagic program; for example, resveratrol can attenuate hepatic steatosis via cyclic adenosine monophosphate (cAMP) signaling [15]. Thus, herein, we hypothesized that DHM might attenuate the progression of hepatic fibrosis by regulating HSCs activation associated with autophagy. On the other hand, the role of regional immunity in tissue homeostasis has been emphasized. Imbalanced regional immunity results in inflammatory injury and even fibrosis in the target organ. Natural killer (NK) cells are vital parts of regional immunity and participate in various liver diseases [16]. Studies have found that NK cells are key players in the process of liver fibrosis [17, 18]. The antifibrotic effect of NK cells is due to the secretion of cytokines such as interferon-gamma (IFN-γ) [19, 20], which can effectively kill activated HSCs (aHSCs). Interestingly, some plant compounds have exhibited various bioactivities, including immunoregulatory effects on NK cells [21, 22]. Thus, we investigated whether DHM can promote hepatic NK cells to eliminate aHSCs, leading to amelioration of liver fibrosis.

In this study, we demonstrated for the first time that DHM can ameliorate the progression of liver fibrosis via inhibition of HSCs activation by inducing autophagy and enhance NK cell-mediated killing of HSCs through IFN-γ secretion. These data offer original insight into the crosstalk between HSCs and hepatic NK cells in the preventive and therapeutic benefits of DHM on liver fibrosis, which probably opens a new door for the development of therapeutic strategies for liver fibrosis.

## Methods

### Chemicals and reagents

DHM was provided by Mansite Bio-Technology (China) and Sigma-Aldrich (USA). CCl_4_ was obtained from Sinopharm Group (China). Transforming growth factor-beta 1 (TGF-β1) was obtained from PeproTech (NJ, USA). 3-Methyladenine (3-MA), bafilomycin A 1 (BafA1), and anti-microtubule associated protein 1 light chain 3 beta (MAP1LC3B/LC3B) antibodies were acquired from Sigma-Aldrich (St. Louis, USA). Antibodies against α-smooth muscle actin (α-SMA), desmin, BECLIN1, signal transducer and activator of transcription 3 (STAT3), and phosphorylated STAT3 (P-STAT3) were obtained from Abcam (UK). Antibodies against sequestosome 1 (SQSTM1), ATG3, nuclear factor kappa-B (NF-κB), and phosphorylated NF-κB p65 (P-NF-κB p65) were purchased from Cell Signaling Technology (USA).

### Animals and experimental procedures

Male C57BL/6 J mice obtained from Third Military Medical University were fed in a controlled environment with a temperature of 22–25 °C and humidity of 50 ± 5% on a 12 h light–dark cycle. Food and water were changed every 3 days and were provided ad libitum. After 1 week, twenty-four 8-week-old mice were randomly grouped into four groups (n = 6): the control, CCl_4_, CCl_4_ + DHM, and DHM groups. To establish the animal model of liver fibrosis, mice were intraperitoneally injected with CCl_4_ (diluted at 1:9 in olive oil) three times a week at the dosage of 2 mL /kg body weight for 6 weeks [23]. DHM was administered by gavage once a day at a dose of 100 mg/kg body weight. After DHM administration, all mice were starved for 12 h and were then anesthetized with pentobarbital sodium before sampling. Serum and liver tissues were collected and stored frozen at -80 °C. All efforts were made to minimize animal suffering. The animal experiments were approved by the Animal Care and Use Committee of Third Military Medical University (Chongqing, China; Approval SYXC-2015-00169).

### Biochemical parameters

To evaluate the liver function, enzymatic assays were conducted to quantify serum alanine aminotransferase (ALT) and aspartate aminotransferase (AST) using a colorimetric spectrophotometer (Roche, Germany) according to the manufacturer’s instructions.

### Histological assessment

Immediately after animals were sacrificed, liver samples were fixed with 4% paraformaldehyde and embedded in paraffin. Then, we sliced the samples into 5-μm-thick sections. To evaluate the microstructure of liver fibrosis, hematoxylin and eosin (H&E) and Sirius Red staining were performed on the liver sections for histological analysis. Images were visualized with a light microscope (Leica, Germany).

### Cell culture and treatment

NK92 cells, a human NK-like cell line, and exclusive complete medium (BNCC342313) were obtained from Beina Chuanglian Biology Research Institute (Beijing, China). LX2 cells (ICell Bioscience Inc., China), a human HSCs line, were cultured in RPMI 1640 medium supplemented with 10% fetal bovine serum (FBS, Gibco) and 1% penicillin–streptomycin at 37 °C in a 5% CO_2_ atmosphere. To establish the model of HSCs activation, LX2 cells were treated with a series of concentrations (0, 2.5, 5, 7.5, and 10 ng/mL) of TGF-β1 for 24 h. To explore the effect of DHM on TGF-β1-treated cells, LX2 and NK92 cells were pretreated with DHM at different concentrations (0, 10, 30, and 50 μM) for 2 h and were then exposed to TGF-β1 (5 ng/mL) for 24 h. Furthermore, to explore the downstream pathway of NK cells in response to DHM, NK92 cells were pretreated with CH223191 (10 μM) for 1 h to inhibit AhR, with stattic (2 μM) for 24 h to inhibit STAT3, or with PDTC (50 μM) for 1 h to inhibit NF-κB. All experiments were repeated at least three times.

### Immunofluorescence analysis

For immunofluorescence of aHSCs in vivo, immunofluorescent staining was performed using anti-α-SMA antibody (Abcam, UK). In short, liver samples were fixed and permeabilized. We incubated the sections with proteinase K (1:200, Solarbio, China) for 30 min, subsequently with Triton X-100 (0.1%, Beyotime, China) for 30 min and with normal goat serum (ZSGB-BIO, China) for 30 min. Next, the primary antibodies were applie at 4℃ overnight, and then secondary antibody for 1 h at room temperature. Finally, DAPI solution (0.1%, Beyotime, China) was applied for 10 min at room temperature.

For immunocytochemistry of HSCs activation in vitro, LX2 cells were seeded on coverslips (NEST, China) in 6-well plates overnight and treated with or without DHM (30 μM) for 2 h. TGF-β1 (5 ng/mL) was added for 24 h to induce the HSCs activation. Subsequently, the cells were washed and fixed. Then, the cells were permeabilized, blocked and stained as mentioned above. Fluorescence images were acquired with an upright microscope (Nikon Eclipse C1).

### Flow cytometry (FCM) analysis

Dissociated cells from the mice were instantly immunostained for FCM analysis to evaluate the frequency of the activation of NK cells. Mononuclear cells were extracted from liver samples with Percoll (Sigma-Aldrich, USA) [24]. The cells were harvested and washed with PBS buffer. The antibodies were used to identify the immune phenotype (Table 3). Cells were stained for 30 min at 4 °C. Data were acquired on a BD FACSVerse flow cytometer (BD Biosciences, USA) and analyzed with FlowJo (v10.7, Becton, Dickinson & Company).

### Coculture of NK cells and HSCs

To explore the effect of NK cells on HSCs supplemented with or without DHM, NK92 cells stimulated with DHM were collected and then cocultured with LX2 cells in a 12-well plate at an E:T ratio of 1:1 for 24 h. For coculture in the Transwell chambers, NK92 cells were plated in the upper chambers, whereas LX2 cells were plated in the lower chambers, which was separated from the upper chambers by filters with a 0.4 μm pore diameter (Corning Life Science, USA). For IFN-γ blockade coculture experiments, DHM-treated NK92 cells were cocultured with LX2 cells in medium containing 4 μg/mL anti-IFN-γ Nab.

### Apoptosis and cell viability assays

To explore the HSCs apoptosis caused by NK cells, an annexin-V/fluorescein isothiocyanate (FITC) apoptosis detection kit (Beyotime Biotech, Beijing, China) was used to evaluate apoptosis according to the manufacturer’s protocol. Briefly, LX2 cells were washed and incubated with annexin V-FITC and propidium iodide (PI) for 20 min in the dark. The samples were examined in a FACScan flow cytometer (Becton Dickinson, Franklin Lakes, NJ, USA). The total apoptotic cell frequency was calculated as the sum of the cell frequencies in the lower right quadrant (apoptotic cells) and the upper right quadrant (necrotic cells).

For adherent cells, a Cell Counting Kit-8 (CCK8, Dojindo, Japan) was used to test cell viability according to the manufacturer’s protocol. Briefly, NK92 cells were replated at a density of 1000 cells/well in 96-well plates. Then, the cells were supplemented with DHM (0, 10, 30 and 50 μM) and TGF-β1 (0 and 5 ng/mL). Aliquots of CCK-8 solution (10 μL; Dojindo, Japan) were incubated at 37 °C for 2 h and optical density (OD) value was measured at 450 nm with a microplate reader (Bio-Rad Laboratories, USA) (n = 3).

### Enzyme-linked immunosorbent assay (ELISA)

α-SMA, Col-1α1, and IFN-γ in the supernatant of cell culture medium were detected using the corresponding commercial ELISA kits (Enzyme-linked Biotechnology, Shanghai, China) according to the manufacturer's instructions. The OD was measured in a SpectraMax® M2 spectrophotometer (Molecular Devices Corp., USA) set at 450 nm.

### Quantitative real-time polymerase chain reaction (qRT-PCR)

RNA was extracted with TRIzol reagent. qRT-PCR was performed in a qTower 2.2 real-time PCR system (Analytik Jena, Germany) using SYBR Premix Ex Taq II (Tli RNaseH Plus, Takara Bio, Japan). All primers are listed in Table 1 (Sangon Biotech, China). Each specimen was analyzed in triplicate, and three PCR runs were conducted for each gene. Relative mRNA expression levels were normalized to those of β-actin and calculated by the 2^−ΔΔCt^ method.

**Table 1:** Sequences of primers used in qRT-PCR

Target gene	Primers
*Col-1a1*	F: 5′-ACTTCCCTACCCAGCACCTT-3′ R: 5′-TGGAGTTGGCACTCTCTCCT-3′
*Col-1a2*	F: 5′-CTCATACAGCCGCGCCCAGG-3′ R: 5′-AGCAGGCGCATGAAGGCGAG-3′
*TIMP-1*	F: 5′-TGCAACTCGGACCTGGTTAT-3′ R: 5′-GAGCAGGGCTCAGATTATGC-3′
*IFN-γ*	F: 5′-GCATCGTTTTGGGTTCTCT-3′ R: 5′-CGCTACATCTGAATGACCTG-3′
*AhR*	F: 5′-TGTGATGCCAAAGGAAGAA -3′ R: 5′-GGGACTCGGCACAATAAA -3′
*CYP1A1*	F: 5′-GGCCACTTTGACCCTTACAA-3′ R: 5′-CAGGTAACGGAGGACAGGAA-3′
*β-actin*	F: 5′-CGAGGCCCCCCTGAAC-3′ R: 5′-GCCAGAGGCGTACAGGGATA-3′

### Western blot analysis

Protein expression was investigated by western blot analysis. Briefly, cells were harvested and lysed with RIPA buffer (Sigma-Aldrich, St. Louis, MO, United States) in the presence of a protease inhibitor mixture (Sigma-Aldrich, St. Louis, MO, United States). The protein content was quantified and extracts were stored at – 80 °C. Proteins (50 μg) were separated and then transferred onto nitrocellulose membranes for 1 h at 220 mA. Membranes were incubated with primary antibodies overnight at 4 °C, followed by secondary antibodies for 1 h. Information on the antibodies used for the above experiments is shown in Table 2. Images were acquired with a Fusion FX imaging system (Vilber Lourmat, France) and quantified and normalized to β-actin bands by densitometry in Quantity One software (version 4.6.2, Bio-Rad) (Table 3).

**Table 2:** Antibodies used for the western blot experiments

Antibody	Dilution	Supplier	Charge number
LC3B	1:1000	Sigma-Aldrich	L7543
SQSTM1	1:1000	CST	5114
NF-κB p65	1:1000	CST	8242
P-NF-κB p65	1:1000	CST	3033 T
ATG3	1:1000	CST	3415
Desmin	1:1000	Abcam	32362 s
STAT3	1:5000	Abcam	119352
P-STAT3	1:2000	Abcam	76315
α-SMA	1:10,000	Abcam	124964
BECLIN1	1:2000	Abcam	207612
β-Actin	1:1000	SANTA Cruz	47778

**Table 3:** Antibodies used for flow cytometric analysis

Antibody	Supplier	Charge number
NK1.1-PE	Biolegend	108707
CD3ε-FITC	Biolegend	100306
NKG2D-APC	Biolegend	130212
IFN-γ-BV421	BD Horizon	563376
BV421 Rat IgG1, κ Isotype	BD Horizon	562868

### Statistical analysis

Statistical analysis was conducted with one-way analysis of variance by SPSS (v13.0, IBM, USA). A two-sided p-value < 0.05 was regarded as statistically significant, and the Tukey–Kramer post hoc test was applied if p < 0.05. Quantitative data are presented as the mean ± standard error of mean (X ± SEM) values. All experiments were repeated independently at least three times.

## Results

### DHM administration ameliorated CCl_4_-induced liver fibrosis and HSCs activation in vivo

**Fig. 1:**
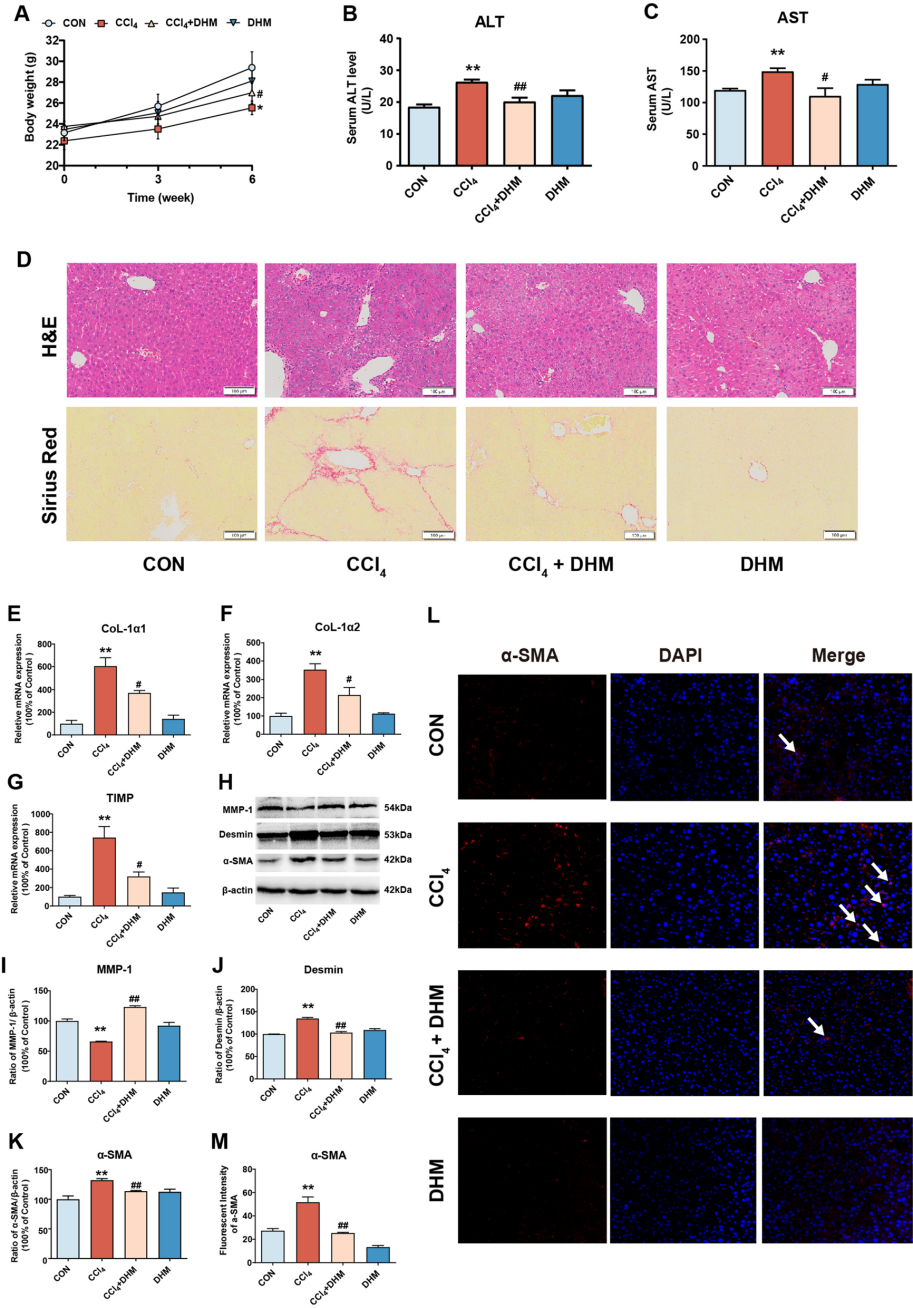
DHM administration ameliorated CCl_4_-induced liver fibrosis and HSCs activation in vivo*.* **a** Body weight was recorded. **b**–**c** Serum levels of aspartate transaminase ALT and AST. **d** Liver fibrosis was examined by H&E and Sirius Red stainings. **e**–**g** The mRNA expressions of CoL-1α1 (**e**), CoL-1α2 (**f**) and TIMP-1 (**g**) were detected by qRT-PCR. **h** The protein levels of MMP1, desmin, and α-SMA were analyzed by western blot. **i**–**k** The quantification of MMP-1 (**i**), desmin (**j**), and α-SMA (**k**) were displayed by histogram, respectively. Data were presented as mean ± SEM (n = 3); **l** HSCs activation was visualized by immunofluorescence assay of α-SMA. **m** The quantification of α-SMA by immunofluorescence assay were displayed by histogram. Data were presented as mean ± SEM (n = 3); *p < 0.05, **p < 0.01, compared to the control group; ^#^p < 0.05, ^##^p < 0.01, compared to the CCl_4_ group. ALT, alanine transaminaseT; AST, aspartate transaminase; α-SMA, α-smooth muscle actin; CCl_4_, carbon tetrachloride; CoL-1α1, collagen I alpha 1; CoL-1α2, collagen I alpha 2; DHM, dihydromyricetin; H&E, hematoxylin and eosin; HSCs, hepatic stellate cells; MMP-1, metalloproteinase 1; TIMP-1, tissue inhibitor of metalloproteinases 1; qRT-PCR, quantitative real-time polymerase chain reaction

To explore the effect of DHM on liver fibrosis, DHM was administered orally to CCl_4_-treated mice, an animal model of liver fibrosis, according to previous study [12]. The body weight of CCl_4_-treated mice was decreased compared with that of control mice (p < 0.05, Fig. 1a), and the serum levels of ALT and AST in mice in the CCl_4_ group were significantly elevated compared with those in mice in the control group (Fig. 1b and c). As expected, the CCl_4_-induced fibrotic effect was significantly inhibited after DHM administration (Fig. 1a–c). Histologically, CCl_4_-induced inflammation and collagen deposition in the liver were obviously ameliorated by DHM administration, as evidenced by H&E and Sirius Red staining (Fig. 1d). Moreover, the CCl_4_-induced increase in the expression of markers of subsequent ECM deposition and fibrosis, including collagen I alpha 1 (CoL-1α1), collagen I alpha 2 (CoL-1α2), and tissue inhibitor of metalloproteinases 1 (TIMP-1), was strongly inhibited by DHM administration (Fig. 1e–g). As reported previously, HSCs activation plays a key role in liver fibrosis, as verified by the high expression of the molecules related to HSCs activation, such as α-SMA [25]. Then, western blot analysis revealed that DHM treatment markedly reversed the CCl_4_-induced elevation in the expression of the fibrosis-related proteins desmin and the decline of matrix metalloproteinase 1 (MMP1) (Fig. 1h–j). Surprisingly, DHM administration led to a significant decrease in α-SMA expression, indicating that DHM could effectively inhibit the CCl_4_-induced HSCs activation (Fig. 1h and k). This result was verified by immunofluorescence staining of α-SMA in the liver (Fig. 1l and m).

In brief, DHM administration notably attenuated liver injury and fibrosis in CCl_4_-treated mice, an effect that was possibly related to the inhibition of HSCs activation.

### DHM treatment inhibited TGF-β1-induced HSCs activation in vitro

**Fig. 2:**
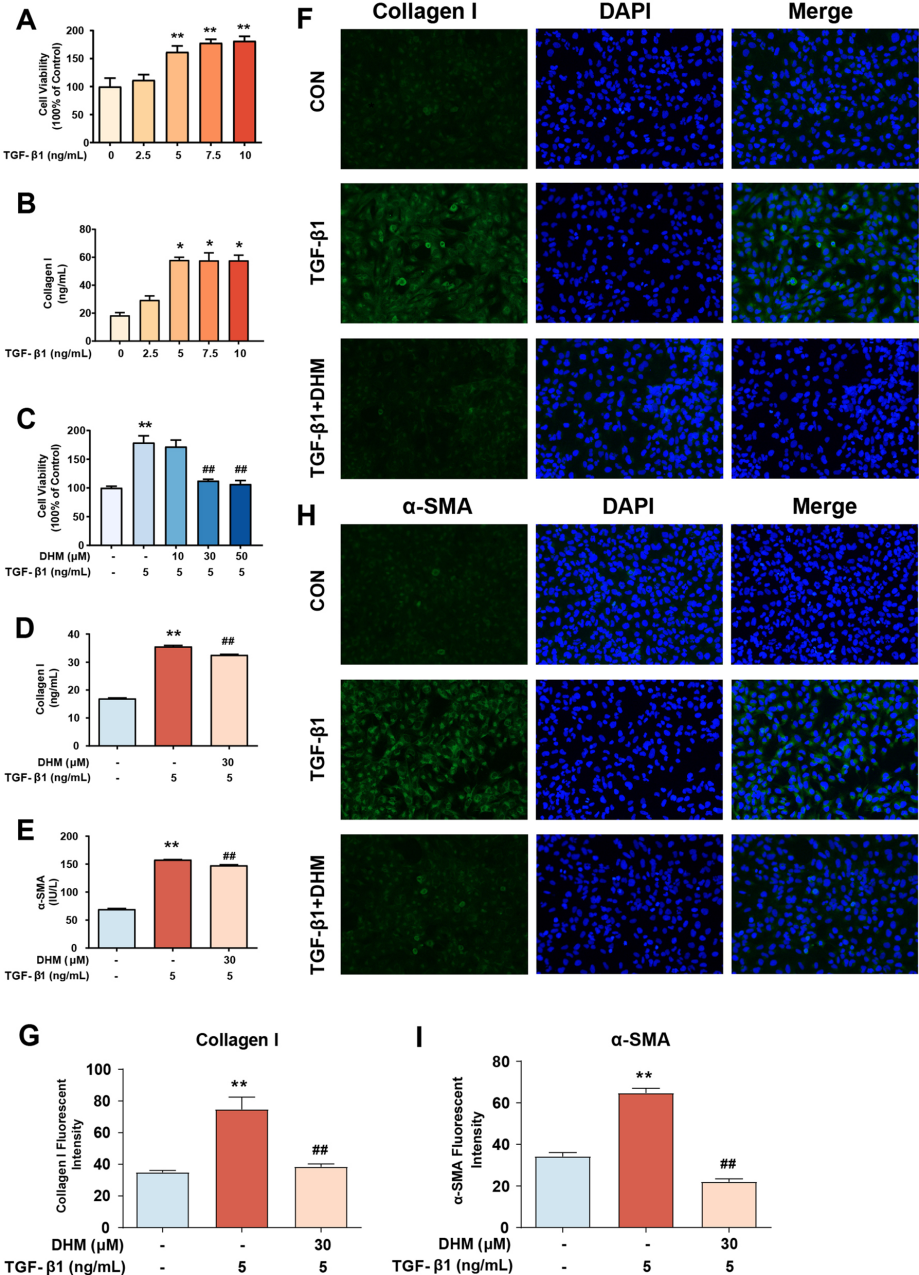
DHM treatment inhibited TGF-β1-induced HSCs activation in vitro*.* **a** Cell viability of LX2 treated by TGF-β1 was detected by CCK-8. **b** The level of collagen I in the cell supernatant was detected by ELISA kit. **c**–**e** After pretreated with DHM for 2 h and followed by TGF-β1 (5 ng/ml) treatment for 24 h, the cell viability of LX2 was determined by CCK-8 assay (**c**), and the levels of collagen I (**d**) and α-SMA (**e**) in cell supernatant were detected by the corresponding ELISA kit. **f**–**g** Representative images of collagen I immunofluorescence staining in LX2 cells. Green fluorescence represents the expression of collagen I and blue fluorescence with DAPI (staining represent cell nucleus) (**f**). The bar charts show the quantification of fluorescence intensity of collagen I (**g**). **h**–**i** Representative images of α-SMA immunofluorescence staining in LX2 cells. Green fluorescence represents the expression of α-SMA and blue fluorescence with DAPI staining represents cell nucleus (**h**). The bar charts show quantification of fluorescence intensity of α-SMA (**i**). Data were presented as mean ± SEM (n = 3); *p < 0.05, **p < 0.01, compared to the control group; ^##^p < 0.01, compared to TGF-β1 group. CCK-8, Cell Counting Kit-8; DAPI, 4′,6-diamidino-2-phenylindole; ELISA, enzyme-linked immunosorbent assay; TGF-β1, transforming growth factor-beta 1

To further explore the role of DHM in HSCs inactivation, we investigated the effect of DHM on LX2 cells, which are characterized as an activated HSC cell line, in vitro*.* To establish a cell model of HSCs activation, LX2 cells were exposed to a series of concentrations (0, 2.5, 5, 7.5, and 10 ng/mL) of TGF-β1 for 24 h. The viability of LX2 cells treated with TGF-β1 was significantly increased compared to that of control cells when the concentration of TGF-β1 was greater than 5 ng/mL (Fig. 2a). Similarly, treatment with TGF-β1 at concentrations greater than 5 ng/mL resulted in notably elevated expression of collagen I, a major collagen protein produced by aHSCs, in LX2 cells (Fig. 2b). Next, LX2 cells were preincubated with a series of concentrations (0, 10, 30, and 50 μM) of DHM for 2 h prior to treatment with TGF-β1 (5 ng/mL) for 24 h. Compared with treatment with TGF-β1 alone, treatment with DHM at concentrations greater than 30 μM significantly suppressed the TGF-β1-induced increase in the viability of LX2 cells (Fig. 2c). Moreover, marked turnover of the aHSC indicators collagen I and α-SMA after DHM treatment was observed by ELISA (p < 0.01), indicating disruption of fibrosis progression (Fig. 2d, e). In addition, similar results were visualized by fluorescence microscopy. These results showed that treatment with 30 μM DHM effectively reduced the expression of collagen I and α-SMA proteins in TGF-β1-treated LX2 cells (Fig. 2f–i).

Taken together, these results indicate that DHM treatment inhibits TGF-β1-induced LX2 activation, suggesting that DHM could inhibit HSCs activation or eliminate aHSCs.

### DHM inhibited HSCs activation by inducing autophagy

**Fig. 3:**
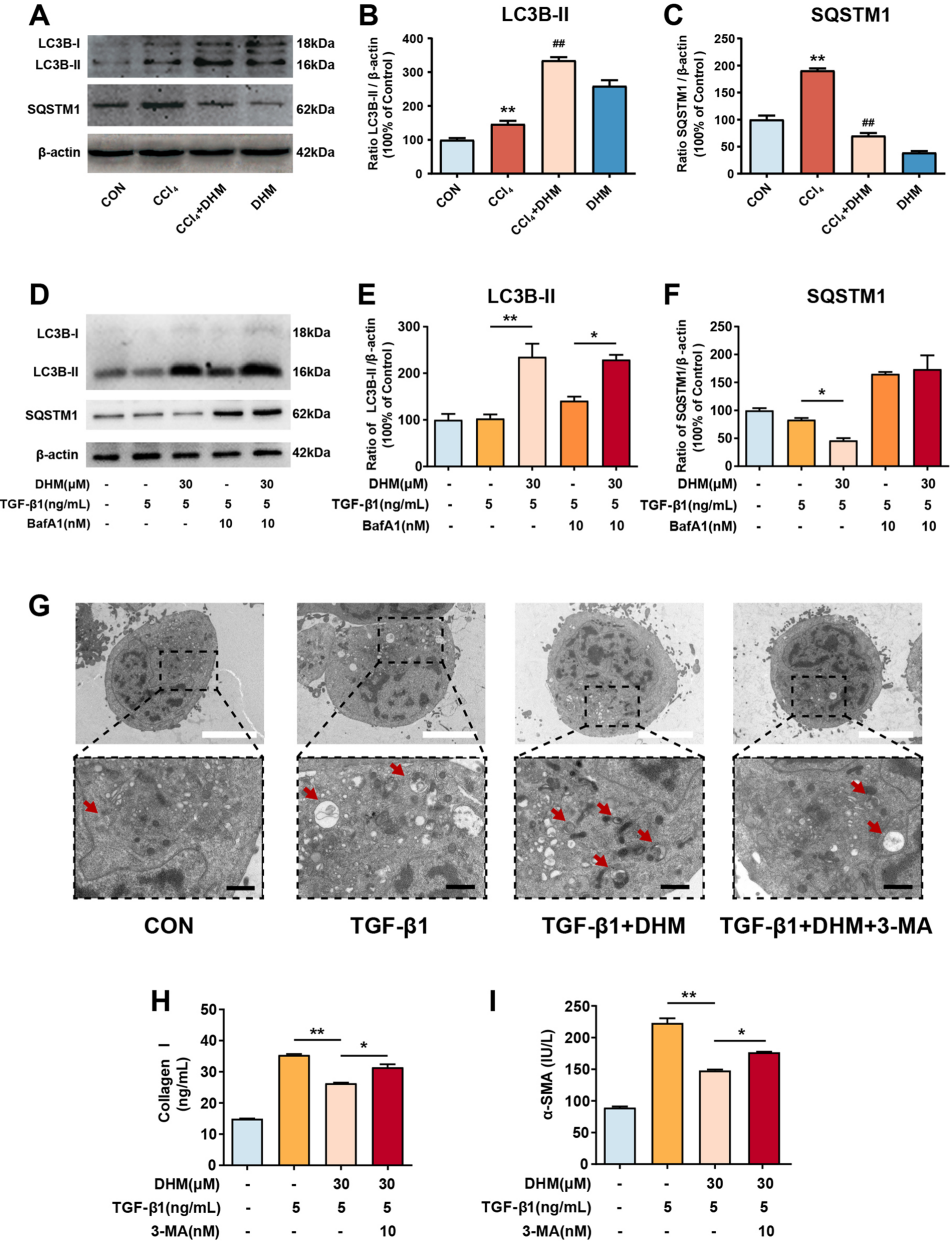
DHM inhibited HSCs activation by inducing autophagy. **a** The protein levels of LC3B-II and SQSTM1 in the mouse liver were analyzed by western blot. **b**–**c** The quantification of LC3B-II (**b**), and SQSTM1 (**c**) were displayed by histogram, respectively. Data were presented as mean ± SEM (n = 3); **d** HSCs were treated with DHM (30 μM) for 2 h, then cells were exposed to TGF-β1 (5 ng/mL) for an additional 24 h. BafA1 (10 nM) was added 1 h before DHM treatment. The expressions of LC3B-II and SQSTM1 were detected by western blot. **e**–**f** Bar charts show the quantification of endogenous LC3B-II (**e**) and SQSTM1 (**f**). **g** LX2 cells were pretreated with DHM (30 μM) in the presence or absence of 3-MA (5 mM) for 2 h, followed by treatment with TGF-β1 (5 ng/mL) for another 24 h. Representative (TEM) images of LX2 cells after treatment. Red arrows indicate the autophagosomes. **h**–**i** Cells were treated as described in (G), and the levels of collagen I (**h**) and α-SMA (**i**) in the medium were detected via ELISA assay. Data were presented as mean ± SEM (n = 3); *p < 0.05, **p < 0.01, compared between the marked groups. 3-MA (3-methyladenine); BafA1, bafilomycin A1; LC3B-II, microtubule associated protein 1 light chain 3 beta; SQSTM1, sequestosome 1; TEM, transmission electron microscopy; TGF-β1, transforming growth factor-beta 1

A previous study revealed that autophagy plays a role in liver fibrosis [14]. To investigate whether autophagy was involved in DHM-induced HSCs inactivation, we measured the hepatic expression levels of autophagic markers in vivo. The result showed that DHM could significantly increase the expression of MAP1LC3B2 (LC3B-II) in the liver from CCl_4_-treated mice. Besides, CCl_4_ could elevate the expression of sequestosome 1 (SQSTM1) in the liver, which was significantly reversed by DHM in vivo as expected (Fig. 3a–c). This result demonstrated that autophagy may be involved in the regulation of DHM on aHSCs activation. To better reveal the precise role of autophagy in the antifibrotic effect of DHM, LX2 cells were used to conduct an in vitro study. LC3B-II, an accurate indicator of autophagic flux, was evaluated using western blotting. DHM pretreatment significantly increased the protein level of LC3B-II in LX2 cells compared with that in the TGF-β1 group (Fig. 3d, e). SQSTM1 is a ubiquitin-binding protein related to cellular signal transduction, oxidative stress and autophagy [26]. Therefore, detection of SQSTM1 is important for evaluating autophagy and autophagy-related diseases. As expected, there was a significant decrease in the SQSTM1 protein level after DHM (30 μM) treatment in TGF-β1-induced LX2 cells (Fig. 3d and f). As well, the expressions of some other autophagic markers such as BECLIN1 and ATG3 were also significantly elevated by DHM in the TGF-β1-treated LX2 cells (Additional file 2: Fig. S2). BafA1 inhibits organelle acidification and autophagosome-lysosome fusion. Thus, we further monitored the LC3B-II level in the presence of BafA1 to evaluate autophagic flux. BafA1 challenge caused LC3B-II accumulation, and the level of LC3B-II was further increased after DHM treatment in TGF-β1-induced LX2 cells (Fig. 3d–f). These results indicate that DHM can initiate autophagic processes in LX2 cells. Moreover, transmission electron microscopy (TEM) was applied to qualify and quantify autophagy [27]. More vacuoles were observed in LX2 cells after DHM treatment, while the number of vacuoles was reduced by treatment with 3-MA, a well-known inhibitor of autophagy in the early phase (Fig. 3g), indicating that DHM-induced autophagy was suppressed by 3-MA treatment. Furthermore, to confirm whether autophagy is associated with DHM-induced downregulation of fibrosis-related markers and inactivation of LX2 cells, the levels of collagen I and α-SMA were measured by the corresponding ELISA kits. The DHM-induced decreases in collagen I and α-SMA expression in TGF-β1-treated LX2 cells were abolished in response to 3-MA treatment (Fig. 3h and i).

In brief, these results suggest that DHM treatment prevents HSCs activation, an effect mediated at least partially through initiation of the cellular autophagic process.

### DHM administration promoted the activation of hepatic NK cells in mice

**Fig. 4:**
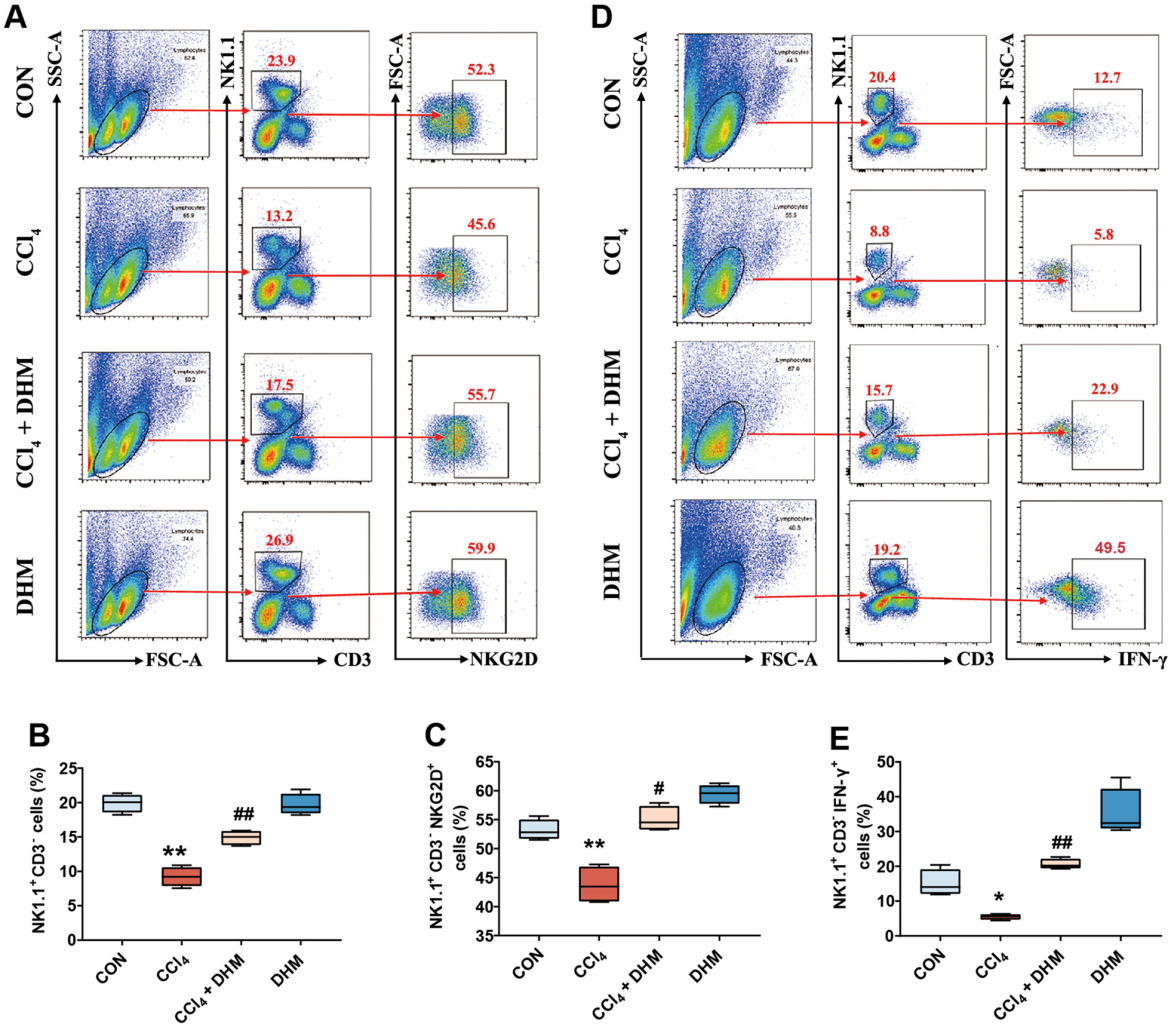
DHM administration promoted the activation of hepatic NK cells in mice. **a**–**c** The frequency of intra-hepatic NK cells (cells were gated by NK1.1^+^ CD3^−^) and NKG2D expression (cells were gated by NK1.1^+^ CD3^−^ NKG2D^+^) which in intra-hepatic NK cells were analyzed by FCM. **d**–**e** Frequencies of intra-hepatic NK cells and IFN-γ expression (cells were gated by NK1.1^+^ CD3^−^ IFN-γ ^+^) in intra-hepatic NK cells were analyzed by FCM. Experiments were independently repeated at least three times. *p < 0.05, **p < 0.01, compared to the control group; ^#^p < 0.05, ^##^p < 0.01, compared to the CCl_4_ group. FCM, flow cytometry; IFN-γ, interferon gamma; NK cells, natural killer cells; NKG2D, NK cell receptor natural killer group 2 member D

Previous studies have reported that hepatic NK cells can exert antifibrotic effects in the liver [28, 29]. However, the NK cell-dependent immune response and antifibrotic activity are suppressed during the progression of liver fibrosis [30]. Thus, we sought to explore the effect of DHM on NK cells throughout the development of liver fibrosis in mice through ex vivo studies. Reasonable control was provided for accurate gating in FCM (Additional file 2: Fig. S1A and B). FCM analysis showed that the frequency of intrahepatic NK cells (NK1.1^+^CD3^−^) declined sharply ex vivo after CCl_4_ treatment in mice (Fig. 4a, b), in line with the results of previous studies [31]. Surprisingly, the frequency of hepatic NK cells (NK1.1^+^CD3^−^) was significantly increased after DHM administration (Fig. 4a, b), indicating that DHM can increase the frequency of hepatic NK cells during liver fibrosis. Furthermore, the frequency of cells expressing NKG2D, an active receptor of NK cells, was significantly increased after DHM administration (Fig. 4a and c). As reported, IFN-γ is a core cytokine related to the NK cell-dependent antifibrotic immune response. Thus, we tried to explore the effect of DHM on the expression of IFN-γ. Interestingly, the frequency of IFN-γ expression was increased after DHM administration compared with that in the CCl_4_ group (Fig. 4d and e).

Collectively, these results demonstrate that the activities of hepatic NK cells in mice can be effectively enhanced by DHM administration.

### DHM treatment enhanced NK cell-mediated killing of HSCs via IFN-γ in vitro

**Fig. 5:**
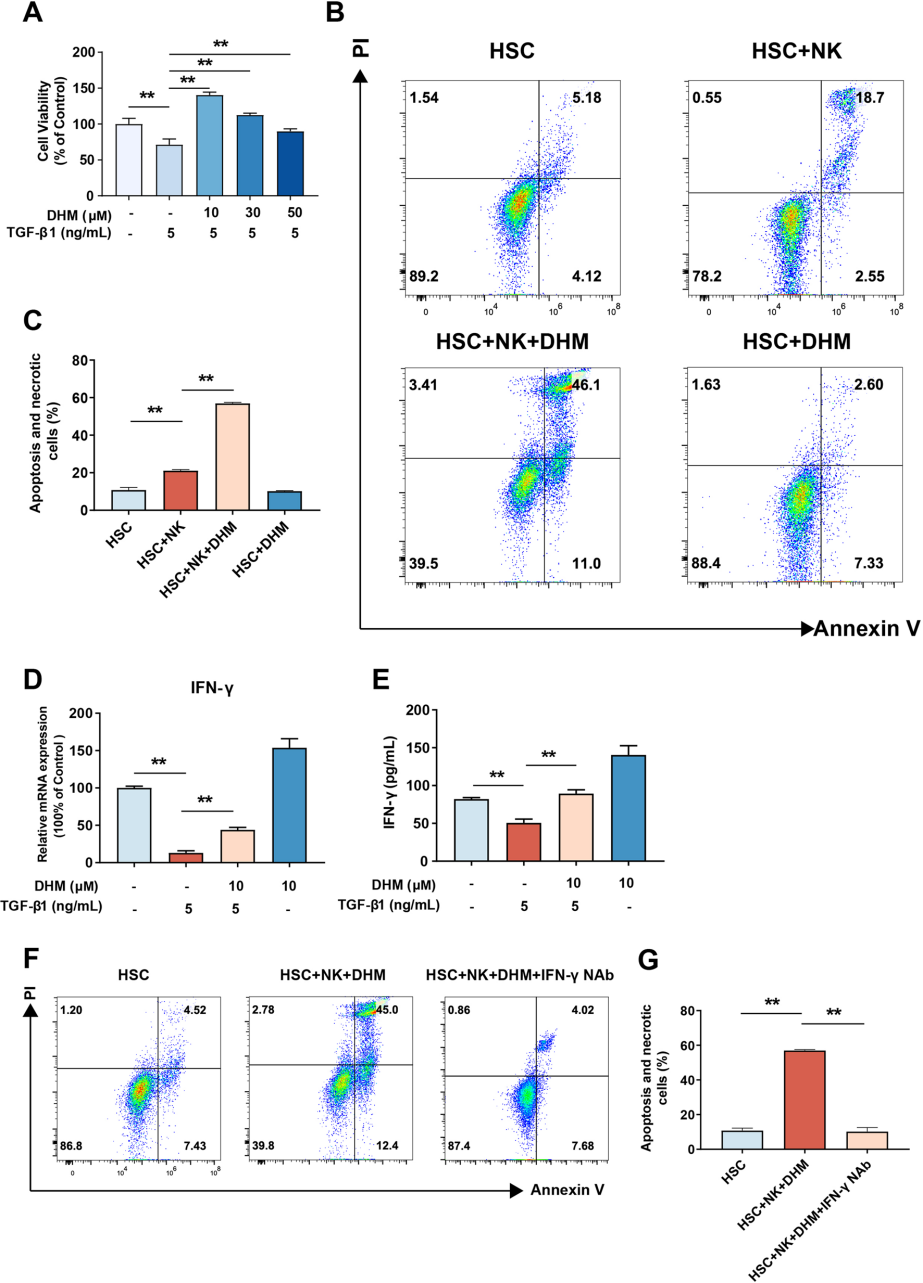
DHM treatment enhanced NK cell-mediated killing of HSCs via IFN-γ in vitro*.* **a** The NK92 cells were treated with DHM in a series of concentrations (0, 10, 30 and 50 μM) for 2 h, followed by the treatment of TGF-β1 (5 ng/ml) for 24 h. Cell viability was detected by CCK-8. **b**–**c** The NK92 cells were pre-incubated with DHM (10 μM) for 2 h and co-cultured with LX2 cells. The PI^±^/Annexin V^+^ population of LX2 was detected by FCM. (D-E) NK cells were pretreated with DHM (10 μM) for 2 h and were exposed to TGF-β1 (5 ng/ml) for 24 h. The level of IFN-γ was quantified by qRT-PCR (D) and ELISA assays (**e**). **f**–**g** Before co-cultured with LX2 cells, NK92 was pretreated with an anti-IFN-γ antibody to neutralize the secreted IFN-γ, then treated with DHM simultaneously. The apoptotic and necrotic percentage of LX2 cells was measured by FCM. Experiments were independently repeated at least three times. *p < 0.05, **p < 0.01, compared between the marked groups

Previous studies have shown that NK cells can kill activated stellate cells to exert antifibrotic effects [20]. Due to the significant effect of DHM on intrahepatic NK cell activation in vivo (Fig. 4), we investigated the effect of DHM on the interaction of NK and HSCs cells in vitro. NK92 cells were preincubated with a series of concentrations (0, 10, 30, and 50 μM) of DHM for 2 h prior to treatment with TGF-β1 (5 ng/mL) for 24 h. Cell viability was determined by CCK-8 assay, and the results showed that compared with TGF-β1 treatment alone, DHM treatment (≥ 10 μM) significantly suppressed the TGF-β1-induced decrease in NK92 cell viability (Fig. 5a). To investigate whether DHM can enhance the immunologic function of NK cells in killing LX2 cells in vitro, NK92 and LX2 cells were cocultured in a transwell chamber and were then treated with or without DHM. Apoptotic and necrotic LX2 cells were then stained with PI and Annexin V staining and evaluated by FCM. As shown in Fig. 5b and c, DHM treatment in the HSCs and NK coculture system resulted in an increased percentage of apoptotic and necrotic cells compared to that in the non-DHM-treated group, indicating that DHM treatment induced NK92 cell-mediated killing of LX2 cells. The positive and negative control for PI and Annexin V staining were also provided (Additional file 1: Fig. S1C). To determine whether IFN-γ plays a critical role in the above biological process, the mRNA expression level and level of secreted IFN-γ in the medium were detected. We found that IFN-γ mRNA expression in and secretion from NK92 cells were significantly inhibited after exposure to TGF-β1, but this inhibition was strongly reversed by DHM treatment (Fig. 5d, e). These results suggested that the increased induction of apoptosis and necrosis of HSCs in the coculture system mediated by DHM treatment might be associated with the DHM-induced increase in IFN-γ secretion from NK cells. To verify this hypothesis, an anti-IFN-γ neutralizing antibody was added to neutralize the secreted IFN-γ before coculture with LX2 cells. When DHM-treated NK cells were pretreated with the neutralizing antibody, the percentage of apoptotic and necrotic LX2 cells was significantly reduced, indicating that IFN-γ was the dominant contributor to the DHM-induced killing effect of NK92 cells on LX2 cells (Fig. 5f, g).

These data indicate that DHM treatment enhances NK cell-mediated killing of HSCs via a mechanism involving the induction of IFN-γ production in NK cells.

### DHM treatment induced IFN-γ production in NK cells through the AhR-NF-κB/STAT3 signaling pathway

**Fig. 6:**
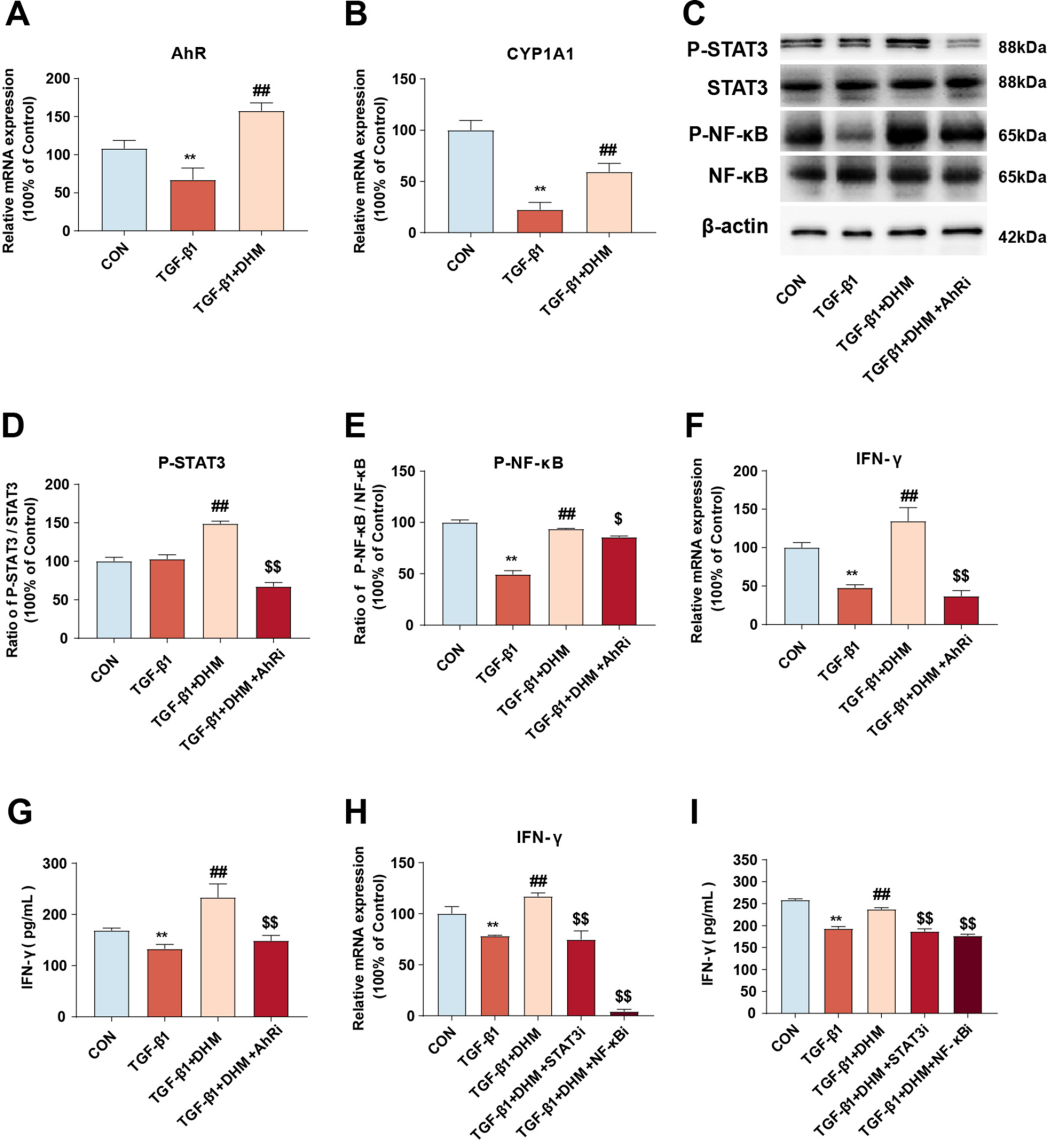
DHM treatment induced IFN-γ production in NK cells through AhR-NF-κB/STAT3 signaling pathway. **a**, **b** NK92 cells were pretreated with DHM (10 μM) for 2 h and were exposed to TGF-β1 (5 ng/ml) for 24 h. The mRNA levels of (AhR) (**a**) and (CYP1A1) (**b**) were quantified by qRT-PCR assay. (C) NK92 cells were preincubated with CH223191 (10 μM) for 1 h to inhibit AhR. Thereafter, cells were treated with DHM (10 μM) for 2 h, then exposed to TGF-β1 (5 ng/ml) for another 24 h. The expressions of P-STAT3, STAT3, P-NF-κB and NF-κB were measured by western blot. **d**, **e** The bar graphs show the quantification of the indicated proteins. **f**, **g** Cells were treated as described in **c**. The mRNA level of IFN-γ was detected by qRT-PCR (**f**) and the expression level of IFN-γ in the medium was detected via ELISA (**g**). **h**, **i** STAT3 and NF-κB were knocked down by the corresponding inhibitor. After that, the cells were pretreated with DHM (10 μM) for 2 h and then incubated with TGF-β1 (5 ng/ml) for an additional 24 h. The bar charts show the mRNA level of IFN-γ by qRT-PCR (H) and the level of IFN-γ by ELISA assay (I). Data were presented as mean ± SEM (n = 3); **p < 0.01, compared to the control group; ^##^p < 0.01, compared to TGF-β1 group; ^$^p < 0.05, ^$ $^p < 0.01, compared to DHM and TGF-β1 co-treated group. AhR, aryl hydrocarbon receptor; CYP1A1, cytochrome P450 family 1 subfamily a polypeptide 1; STAT3, signal transducers and activators of transcription 3

Several studies have shown that the aryl hydrocarbon receptor (AhR) is a key nuclear receptor and transcription factor in some immune cells and is involved in the regulation of a variety of cytokines [32]. AhR is also a natural receptor for several polyphenolic compounds, such as resveratrol and flavonoids [33, 34]. According to some studies, AhR is an upstream molecule of nuclear factor kappa-B (NF-κB) and signal transducer and activator of transcription 3 (STAT3) signaling, which is related to IFN-γ production [35]. Thus, we investigated whether AhR is involved in DHM-induced IFN-γ production in NK cells in vitro. As shown in Fig. 6a and b, NK92 cells were treated with TGF-β1, and the mRNA expression levels of AhR and CYP1A1, a marker of AhR activation, were notably decreased. However, the TGF-β1-induced decreases in the AhR and CYP1A1 expression levels were notably reversed by DHM treatment. These results indicate that DHM can promote the activation of AhR. Then, the phosphorylation of NF-κB and STAT3 was analyzed by western blotting. We found that exposure to TGF-β1 resulted in a decreased P-NF-κB/NF-κB ratio, whereas DHM pretreatment significantly reversed the TGF-β1-induced effects. Additionally, DHM supplementation led to an increased P-STAT3/STAT3 ratio (Fig. 6c-e). However, these benefits of DHM were abolished by treatment with the AhR inhibitor (AhRi) CH223191 (10 μM), implying a critical role of AhR in the regulation of NK cell activation by DHM treatment (Fig. 6c–e). Furthermore, similar results were observed with respect to the mRNA and protein expression levels of IFN-γ in the supernatant (Fig. 6f–g). Moreover, the ELISA and qRT-PCR results showed that the DHM-induced upregulation of IFN-γ expression in TGF-β1-treated NK92 cells was notably eliminated by treatment with a STAT3 inhibitor (2 μM stattic for 24 h) or an NF-κB inhibitor (50 μM PDTC for 1 h), as shown in Fig. 6h and i. Collectively, these results demonstrate that DHM induces IFN-γ production by activating the AhR-NF-κB/STAT3 signaling pathway in NK cells.

## Discussion

**Fig. 7:**
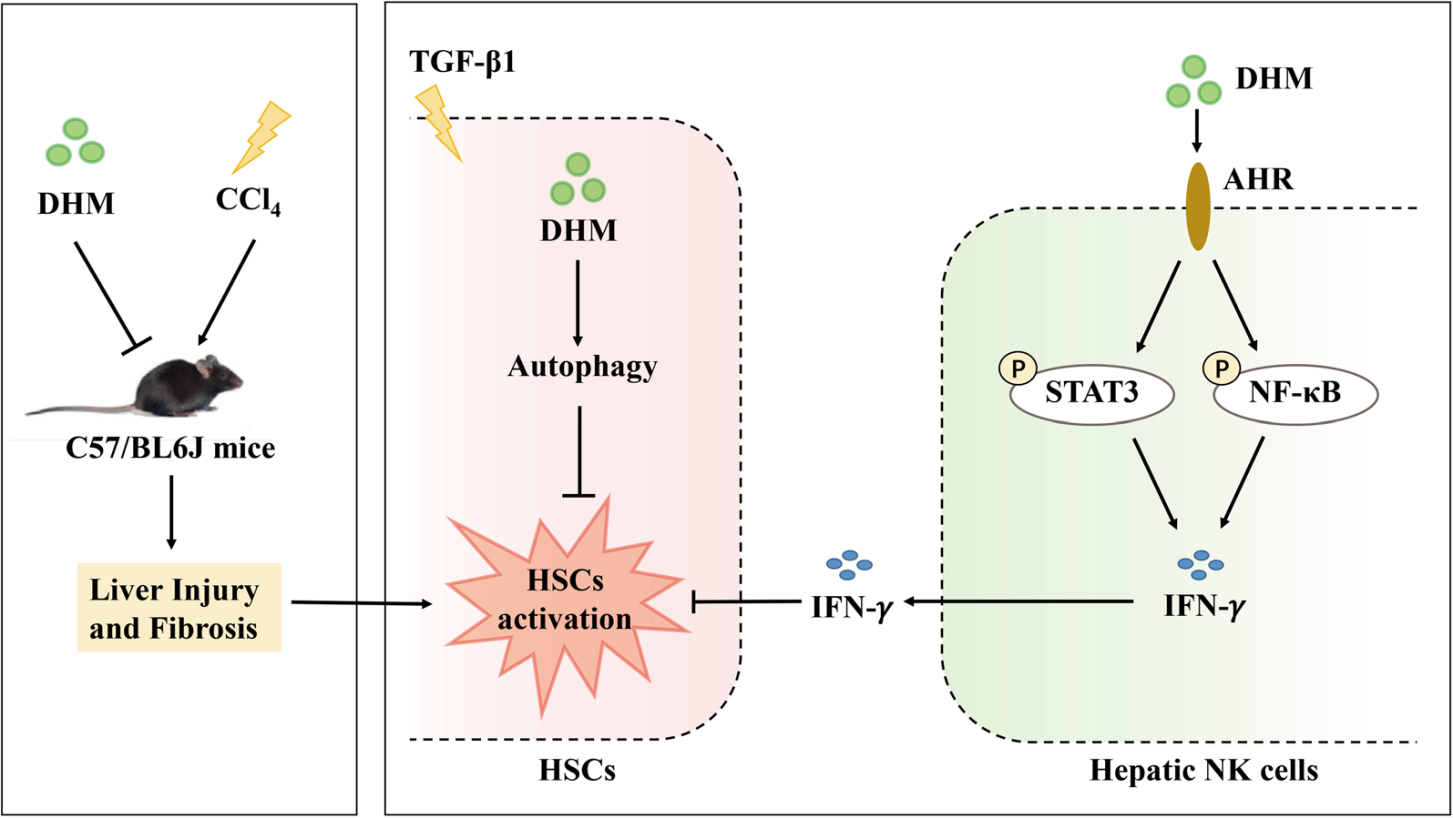
Schematic diagram showing the proposed mechanism of the preventive effect of DHM against liver fibrosis via inhibiting HSCs activation by inducing autophagy and enhanced NK cells killing effect to HSCs through the AhR-NF-κB/STAT3- IFN-γ pathways. DHM attenuated CCl_4_-induced liver injury and fibrosis in vivo. On one hand, DHM-induced benefits were closely associated with the inhibition of HSCs activation by induction of autophagy. And on the other hand, DHM enhanced the killing effect of hepatic NK cells to the activated HSCs through AhR-NF-κB/STAT3-IFN-γ signaling pathway

Suppression of fibrosis is a vital step in preventing the progression of liver diseases and restoring liver homeostasis. However, there is currently no approved therapy for fibrosis suppression. Emerging evidence suggests that aHSCs are the main cells responsible for liver fibrogenesis [13]. In this study, our data indicated that DHM can effectively ameliorate CCl_4_-induced liver fibrosis by drastically inhibiting HSCs activation. The beneficial effect of DHM is mediated via two main routes: direct inactivation of aHSCs via autophagy induction and indirect enhancement of hepatic NK cell-mediated killing of aHSCs via stimulation of IFN-γ production (Fig. 7). Our study demonstrated the prospects of DHM for the development of a novel antifibrotic strategy for the future treatment of liver fibrosis.

HSCs are the predominant cells responsible for the fibrotic process in the liver, because their activation leads to most of the architectural changes that characterize the fibrotic liver. When the liver is damaged, HSCs with the quiescent phenotype (qHSCs) reduce their vitamin A storage and gradually transform into an activated phenotype. aHSCs release cytokines that promote ECM synthesis and deposition, which finally leads to liver fibrosis. Thus, inhibiting aHSCs is a potential strategy to reverse liver fibrosis. However, effective regulatory methods are still being explored. Recently, researchers have suggested that dietary natural polyphenols provided several benefits ameliorating metabolic disorders, including liver diseases [10, 36]. Some natural compounds are available through the diet with limited side effects. Among these compounds, DHM, which is enriched in *Ampelopsis grossedentata*, is a flavonoid historically regarded as a therapeutic herbal medicine for hepatoprotection in China. Some studies have demonstrated that DHM exhibits anti-inflammatory, antioxidative, antihypertensive, lipid regulatory, and antitumor effects [7, 9, 37, 38]. Moreover, some findings have suggested that DHM exerts a protective effect against liver injury [39, 40]. Many researchers have identified the predominant therapeutic targets of DHM as hepatocytes [41]. However, whether DHM exerts an impact on aHSCs inactivation is still uncertain. In this study, we explored the effect of DHM on liver fibrosis in CCl_4_-induced mice in vivo and on TGF-β1-induced HSC activation in vitro. Surprisingly, we discovered that DHM can alleviate liver fibrosis by inhibiting HSC activation (Figs. 1 and 2). Then, we further explored the precise mechanism by which DHM inhibits HSC activation. Interestingly, our study also demonstrated that DHM can regulate the hepatic cellular microenvironment by regulating HSCs and NK cells in the process of liver fibrosis, indicating the versatility of DHM in the liver.

DHM activated the autophagy signaling pathway in HSCs to inhibit their activation directly. Autophagy, the cellular housekeeping program for the removal of injured proteins and organelles through a degradation mechanism, is critical in several pathologies [42–44]. Furthermore, the latest studies have indicated that autophagy plays a key protective role in liver disease. For example, He et al. indicated that the microRNA-125a/VDR axis reduced autophagic flux and subsequently led to fibrosis in a mouse model and in humans [45]. Importantly, a recent study revealed that autophagy is an efficient target in HSCs for attenuation of liver fibrosis [46]. Interestingly, DHM plays an important role in activating autophagy in many organ systems. For instance, DHM can prevent diabetic cardiomyopathy by activating autophagy [47]. Guo et al. reported that DHM triggers autophagy in the renal system [48]. Notably, DHM-induced autophagy alleviates ethanol-induced hepatic injury [49]. Thus, we hypothesized that the hepatoprotective effect of DHM depends on autophagy induction in HSCs. In our study, we found that DHM effectively triggered autophagy and subsequently inhibited the expression of fibrogenic or aHSC markers in a CCl_4_-induced mouse model of liver fibrosis in vivo and suppressed TGF-β1-induced HSCs activation in vitro (Fig. 3 and Additional file 2: Fig. S2). In contrast, 3-MA completely abolished DHM-induced HSCs inactivation, proving the key role of autophagy in the hepatoprotective effect of DHM. In line with previous findings, autophagy has been reported to inhibit extracellular vesicle release from HSCs to alleviate liver fibrosis [46]. In our study, the regulatory role of DHM in autophagy in HSCs could explain its potential antifibrotic mechanism to a certain extent.

Another antifibrotic mechanism of DHM is its immunoregulation of NK cell-mediated aHSCs killing via the AhR-NF-κB/STAT3-IFN-γ signaling pathway. Accumulating evidence has demonstrated that immunoregulation is involved in the microenvironment of liver injury [50–52]. In particular, NK cells are key players in alleviating the fibrotic process [30, 53, 54]. Previously, some plant compounds were shown to exhibit high regulatory activity toward NK cells [21, 55]. Thus, we aimed to examine the roles of DHM in the immunological functions of hepatic NK cells in the process of liver fibrosis. Our results revealed that DHM can increase the activity of hepatic NK cells, as evidenced by the elevated frequency of activated hepatic NK cells (NK1.1^+^CD3^−^NKG2D^+^) after DHM administration in vivo. Moreover, the hepatic NK cells in DHM-treated mice secreted more IFN-γ than their non-DHM-treated counterparts (Fig. 4). IFN-γ, an important cytokine with functions in the elimination of pathogens, remodeling of the immunological function of Th1 helper cells, and enhancement of antigen-presenting cells and MHC class I expression, also exhibits notable antifibrotic capability, as evidenced by the enhanced fibrotic response in CCl_4_-treated mice with IFN-γ blockade. Thus, we aimed to determine whether DHM can regulate IFN-γ expression and secretion by NK cells to inhibit aHSCs. As expected, we found that DHM could drive the activities of NK cells and suppress aHSCs, mainly by inducing IFN-γ production (Fig. 5). In addition, some other studies can support our finding [56, 57]. For example, IFN-γ has been found to induce apoptosis or cell cycle arrest in HSCs [58].

The potential mechanism of DHM-induced IFN-γ production needs to be elucidated. AhR, a highly conserved intracellular transcription factor, is characterized as a ligand-activated transcription factor participating in various physiological processes [59, 60]. Numerous studies have indicated that AhR can strongly regulate the function of NK cells [61, 62]. For instance, resveratrol can significantly enhance NK cell-mediated killing of HSCs by activating AhR [63]. In line with previous studies, our study also found that DHM treatment notably boosted the expression of AhR and IFN-γ in TGF-β1-treated NK cells (Fig. 6). It is worth noting that inhibition of AhR significantly compromised DHM-induced IFN-γ secretion. Collectively, these findings proved that AhR can effectively regulate IFN-γ expression and secretion. Furthermore, mechanistic studies provided in-depth evidence that STAT3 possibly participates in the process of AhR-induced IFN-γ secretion (Fig. 6). This possibility is supported by the work by Shi et al., in which the IL-22/STAT3 signaling pathway was shown to be activated by AhR after indole-3-acetic acid treatment [64]. Similarly, Jie et al. identified that elevated expression and activation of AhR could lead to phosphorylation of STAT3 [65]. Previous studies revealed that another key molecule, NF-κB, is also involved in the process of AhR-induced immunoregulation. For example, AhR forms a complex with STAT1 and NF-κB that regulates LPS-induced inflammatory responses in macrophages [66]. In addition, AhR activation can result in the activation of NF-κB signaling [67]. We found that AhR inhibition led to a decrease in NF-κB. Moreover, inhibition of NF-κB significantly decreased DHM-induced IFN-γ secretion (Fig. 6). Our previous study showed that TAARD-mediated production of IFN-γ by activated NK cells was induced by NF-κB and STAT3 signaling pathway activity mediated by TLR [35]. In addition, phyllanthusmin C has been found to enhance IFN-γ secretion by NK cells via NF-κB signaling [68]. Collectively, these results demonstrate that NF-κB could be another downstream target of AhR in NK cell activation. Taken together, our findings show that DHM-induced IFN-γ production in NK cells is mediated partially through activation of the AhR-NF-κB and AhR-STAT3 signaling pathways.

There are still some limitations of our study. First, part of our data was based on mice with CCl_4_-induced liver fibrosis. Whether DHM inhibits liver fibrosis in other models, such as mice with high-fat diet-induced liver fibrosis, needs to be confirmed in the future. In addition, whether additional mechanisms are involved in the beneficial effects of DHM on HSC activation needs to be investigated.

## Conclusions

In this study, we found for the first time that DHM can attenuate the progression of liver fibrosis by inhibiting HSCs activation directly and indirectly (Fig. 7). These results provide critical experimental evidence indicating the potential efficacy of DHM in future clinical applications for preventing and treating liver fibrosis.

## Supplementary Information

**Additional file 1: Figure 1. The relevant controls for the flowcytometry.** (A) Flow cytometry gating strategies for the FMO control(left) and APC-NKG2D^+^ (right). (B) Flow cytometry gating strategiesfor the isotype control (left) and BV421-IFN-γ^+^ (right). C. Flowcytometry gating strategies for the negative control (left) and positivecontrol (right) for the PI and Annexin V staining. FMO, fluorescence minusonecontrol.

**Additional file 2: Figure 2. DHM treatment triggeredautophagy in TGF-β1-treated HSCs.** (A)LX2 cells were treated with DHM (30 μM) for 2 h, then cells were exposed to TGF-β1 (5 ng/mL) for an additional 24 h. The expressions ofBECLIN1 and ATG3 were detected bywestern blot. (B-C) Bar charts show the quantification of endogenous BECLIN1 (B)and ATG3 (C).

## Data Availability

Access to the data of this study will be considered by the corresponding author upon reasonable request.

## Abbreviations

3-MA: 3-Methyladenine
AhR: Aryl hydrocarbon receptor
aHSCs: Activated hepatic stellate cells (aHSCs)
ALT: Alanine transaminase
AST: Aspartate transaminase
α-SMA: α-Smooth muscle actin
BafA1: Bafilomycin A1
BSA: Bovine serum albumin
cAMP: Cyclic adenosine monophosphate
CCK8: Cell counting kit-8
CCl_4_: Carbon tetrachloride
CCK-8: Cell counting kit-8
CoL-1α1: Collagen I alpha 1
CoL-1α2: Collagen I alpha 2
CYP1A1: Cytochrome P450 family 1 subfamily a polypeptide 1
DAPI: 4′,6-Diamidino-2-phenylindole
DHM: Dihydromyricetin
ECM: Extracellular matrix
ELISA: Enzyme-linked immunosorbent assay
FCM: Flow cytometry
FITC: Fluorescein isothiocyanate
H&E: Hematoxylin and eosin
HSCs: Hepatic stellate cells
IFN-γ: Interferon gamma
LC3: Light chain 3
MAP1LC3B/LC3B: Microtubule associated protein 1 light chain 3 beta
MMP-1: Matrix metalloproteinase-1
mRNA: Messenger ribonucleic acid
NAFLD: Nonalcoholic fatty liver disease
NF-κB: Nuclear factor kappa-B
NK cell: Natural killer cell
NKG2D: NK cell receptor natural killer group 2 member D
qRT-PCR: Quantitative real-time polymerase chain reaction
SQSTM1: Sequestosome 1
STAT3: Signal transducers and activators of transcription 3
TGF-β1: Transforming growth factor-beta 1
TIMP-1: Tissue inhibitor of metalloproteinase-1
TEM: Transmission electron microscopy
