# Supplementary figures and images for: Dihydromyricetin ameliorates liver fibrosis via inhibition of hepatic stellate cells by inducing autophagy and natural killer cell-mediated killing effect
Source: Nutr Metab (Lond). 2021 Jun 19;18:64. doi: 10.1186/s12986-021-00589-6 (PMC8214786; doi:10.1186/s12986-021-00589-6)

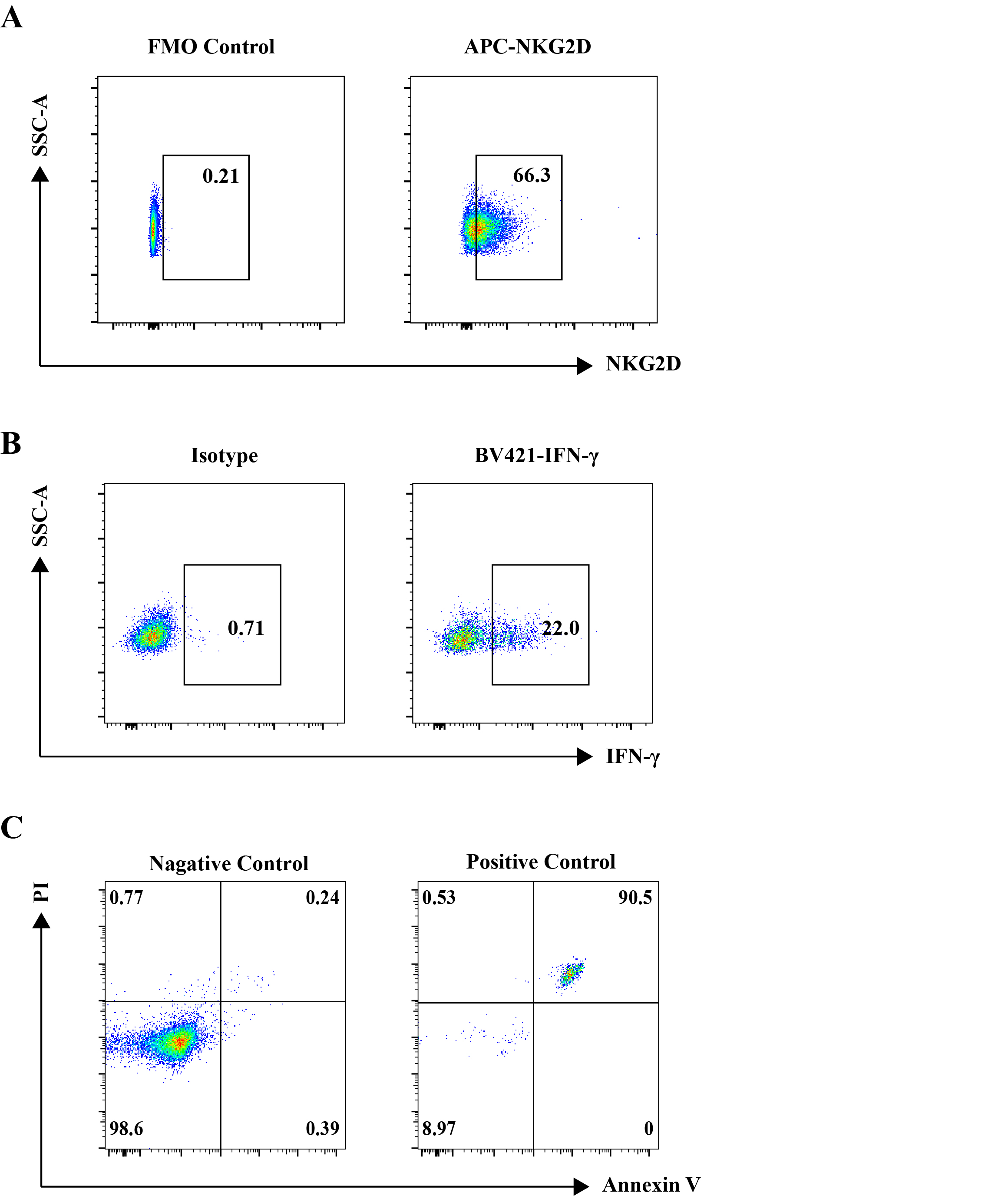

Supplement: Supplementary file 1 — Additional file 1: Figure 1. The relevant controls for the flowcytometry. (A) Flow cytometry gating strategies for the FMO control(left) and APC-NKG2D+ (right). (B) Flow cytometry gating strategiesfor the isotype control (left) and BV421-IFN-γ+ (right). C. Flowcytometry gating strategies for the negative control (left) and positivecontrol (right) for the PI and Annexin V staining. FMO, fluorescence minusonecontrol. [file 12986_2021_589_MOESM1_ESM.jpg]

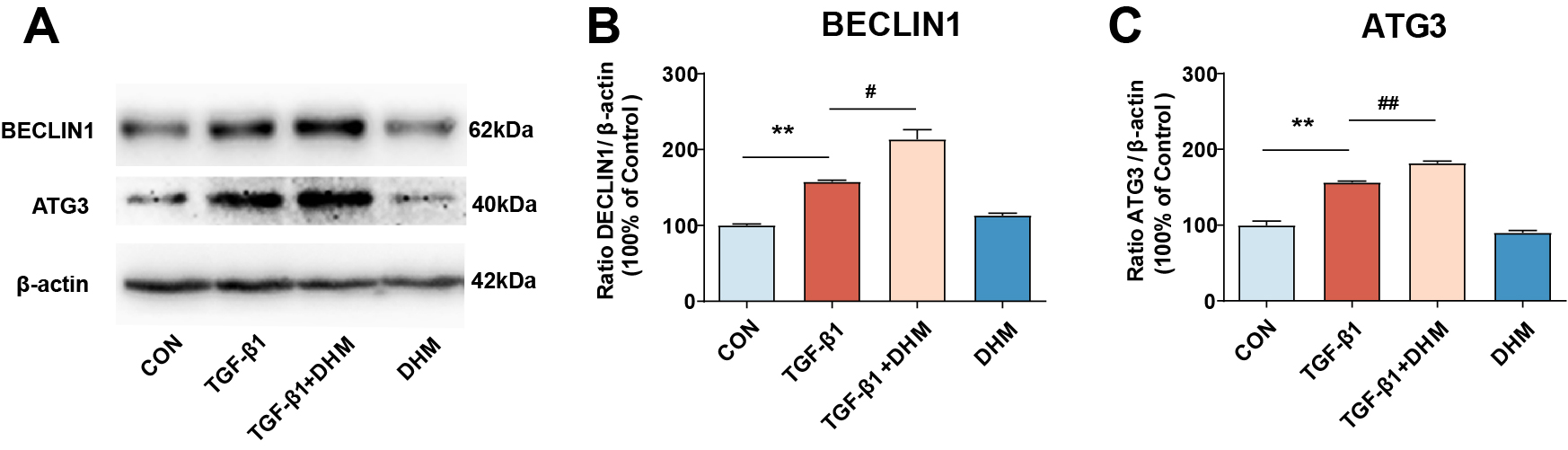

Supplement: Supplementary file 2 — Additional file 2: Figure 2. DHM treatment triggeredautophagy in TGF-β1-treated HSCs. (A)LX2 cells were treated with DHM (30 μM) for 2 h, then cells were exposed to TGF-β1 (5 ng/mL) for an additional 24 h. The expressions ofBECLIN1 and ATG3 were detected bywestern blot. (B-C) Bar charts show the quantification of endogenous BECLIN1 (B)and ATG3 (C). [file 12986_2021_589_MOESM2_ESM.jpg]
